# pH-Responsive Cinnamaldehyde–Arginine Nanoprodrug for Targeted Rheumatoid Arthritis Therapy via Antioxidant Activity and Macrophage Reprogramming

**DOI:** 10.3390/antiox15040469

**Published:** 2026-04-10

**Authors:** Lihong Huang, Wenlong Zhang, Shuai Qiu, Dazhi Yang, Qingyun Tang, Jiajun Huang, Lei Liu, Yang Kang, Shuo Tang

**Affiliations:** 1 Department of Orthopaedics, The Eighth Affiliated Hospital, Sun Yat-Sen University, Shenzhen 518033, China; huanglh65@mail2.sysu.edu.cn (L.H.); chmlung@mail2.sysu.edu.cn (W.Z.); qiush23@mail.sysu.edu.cn (S.Q.); tangqy6@mail2.sysu.edu.cn (Q.T.); huangjj266@mail.sysu.edu.cn (J.H.); liulei56@mail.sysu.edu.cn (L.L.); 2 Scientific Research Center, The Seventh Affiliated Hospital, Sun Yat-Sen University, Shenzhen 518107, China; 3 Department of Spine Surgery, Shenzhen Nanshan Hospital, Shenzhen 518052, China; dazhiyang@email.szu.edu.cn

**Keywords:** rheumatoid arthritis, pH-responsive nanoprodrug, oxidative stress, cinnamaldehyde

## Abstract

Conventional therapies for rheumatoid arthritis (RA) are limited by poor selectivity, insufficient modulation of the oxidative inflammatory microenvironment, and systemic side effects. Oxidative stress and macrophage-driven immune dysregulation represent critical therapeutic targets. Cinnamaldehyde (CA) and arginine (Arg) possess antioxidant, anti-inflammatory, and anti-osteoclastogenic activities, but their poor solubility, instability, and lack of targeting restrict clinical application. Here, we report a pH-responsive cinnamaldehyde–arginine nanoprodrug (Arg-CA NPs), synthesized via Schiff base reaction, that spontaneously self-assembles into uniform nanoparticles capable of acid-triggered dual-drug release. Arg-CA NPs enhanced the solubility and stability of CA, exhibited excellent dispersibility and circulatory stability, and demonstrated intrinsic antioxidant and anti-inflammatory properties. Mechanistically, Arg-CA NPs attenuated intracellular ROS accumulation, preserved mitochondrial function, and reprogrammed macrophages toward an anti-inflammatory M2 phenotype by suppressing hypoxia-inducible factor-1α (HIF-1α), cyclooxygenase-2 (COX-2), and nuclear factor kappa-light-chain-enhancer of activated B cells (NF-κB) signaling. In an adjuvant-induced arthritis (AIA) rat model, Arg-CA NPs selectively accumulated in inflamed joints and significantly alleviated joint swelling, synovial inflammation, cartilage erosion, and bone destruction. These findings identify Arg-CA NPs as a promising redox-active nanoplatform for RA therapy by targeting oxidative stress and immune dysregulation.

## 1. Introduction

Rheumatoid arthritis (RA) is a chronic autoimmune disease that affects 0.5–1% of the global population, leading to progressive joint destruction and placing a substantial burden on healthcare systems and society worldwide [1,2]. Non-steroidal anti-inflammatory drugs, glucocorticoids (GCs), disease-modifying antirheumatic drugs, and biologic agents have been widely applied in RA management [3]. However, their use is limited by significant drawbacks, including gastrointestinal and cardiovascular adverse events, immunosuppression-related complications, metabolic disturbances, hepatotoxicity, and high cost [4,5,6,7]. Novel therapeutic strategies with improved controlled release and reduced systemic toxicity are therefore urgently required.

A hallmark of RA pathology is the abnormal inflammatory microenvironment within affected joints, characterized by hypoxia, elevated reactive oxygen species (ROS), and a mildly acidic pH resulting from excessive lactic acid production and anaerobic glycolysis [8,9,10]. These conditions exacerbate oxidative stress and activate pro-inflammatory signaling pathways such as nuclear factor kappa-light-chain-enhancer of activated B cells (NF-κB), thereby promoting macrophage M1 polarization and osteoclast-mediated bone resorption [11,12,13].

Given the critical role of oxidative stress in RA progression, delivery systems capable of modulating the oxidative inflammatory microenvironment have attracted increasing attention. In particular, naturally derived bioactive compounds with antioxidant and anti-inflammatory activities represent promising candidates for RA therapy. Numerous phytochemicals have been reported to exert potent antioxidant and anti-inflammatory effects by modulating oxidative stress and inflammatory signaling pathways [14]. These natural products are particularly appealing due to their biocompatibility, low toxicity, and multi-targeted biological activities, offering promising potential for chronic inflammatory disorders.

Among them, cinnamaldehyde (CA), a major active component of cinnamon, has shown considerable therapeutic potential in alleviating inflammation through scavenging excessive reactive oxygen species (ROS), suppressing NF-κB activation, and regulating macrophage polarization [15,16,17,18]. However, the clinical application of free CA remains limited by its poor aqueous solubility, instability, and rapid metabolism, which severely hinder its bioavailability and in vivo efficacy [19]. To overcome these limitations, combining CA with bioactive amino acids has emerged as an effective strategy to enhance stability and broaden therapeutic functions. Arginine (Arg), a semi-essential amino acid involved in immune regulation and nitric oxide synthesis, plays a vital role in macrophage function and tissue repair. Arg has been reported to suppress inflammatory signaling by inhibiting the TLR4/NF-κB and MAPK pathways, which regulate stress responses and cytokine expression, and to modulate nucleotide-binding oligomerization domain proteins (NODs), thereby attenuating lipopolysaccharide (LPS)-induced inflammation and preventing cell death [20,21,22]. Therefore, the integration of CA and Arg into a unified nanoplatform is expected to synergistically enhance antioxidant and immunomodulatory properties, while improving the physicochemical stability and targeted delivery of CA, providing a rational design for inflammation-targeted nanotherapy in RA.

**Scheme 1:**
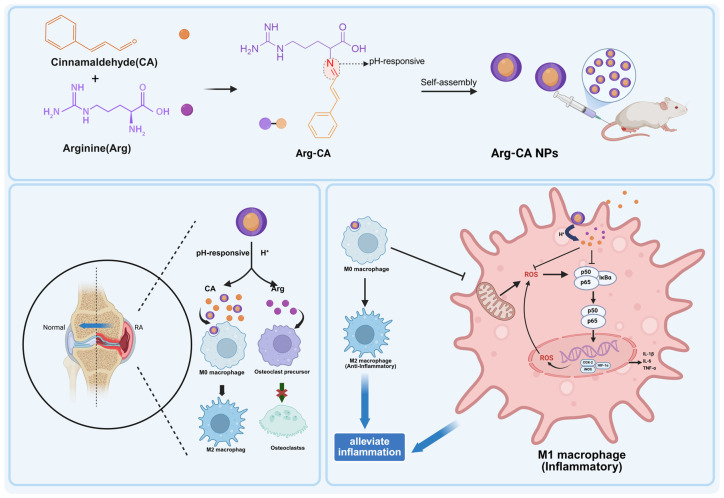
Schematic of the synthesis, delivery, and therapeutic mechanism of Arg-CA nanoparticles for rheumatoid arthritis treatment. Created in BioRender. Yang, S. (2026). https://BioRender.com/p9h6dxr (accessed on 10 march 2026).

In this study, we designed and fabricated a pH-responsive cinnamaldehyde–arginine nanoprodrug (Arg-CA NPs) by condensing the aldehyde group of CA with the amino group of Arg to generate a dual-drug amphiphilic precursor via a Schiff base reaction. Owing to its amphiphilic nature, this precursor spontaneously self-assembled into nanoparticles in aqueous solution. This step enhanced the water solubility and oxidative stability of CA and provided the nanoprodrug with excellent dispersibility and circulatory stability. The acid-labile imine bonds in the Arg-CA NPs enabled selective degradation and dual-drug release in the acidic RA microenvironment. Furthermore, the imine groups conferred additional ROS-scavenging activity. The nanoparticles were systematically characterized, demonstrating well-defined physicochemical properties and acid-triggered drug release. To mimic macrophage activation and joint tissue damage in RA, LPS-stimulated RAW264.7 cells and an adjuvant-induced arthritis (AIA) rat model were employed. Arg-CA NPs exhibited potent ROS-scavenging activity, preserved mitochondrial integrity, and effectively modulated macrophage polarization in vitro. In vivo, Arg-CA NPs markedly reduced joint inflammation, cartilage erosion, and bone destruction. In vivo imaging and biosafety evaluations further revealed preferential accumulation in inflamed joints and excellent biocompatibility. Collectively, these findings identify Arg-CA NPs as a promising redox-active nanoplatform for RA therapy targeting oxidative stress and immune dysregulation. The overall design, delivery process, and proposed therapeutic mechanism of Arg-CA NPs are illustrated in Scheme 1.

Cinnamaldehyde (CA) and arginine (Arg) were conjugated via a Schiff base reaction to form an amphiphilic prodrug (Arg-CA), which self-assembled into pH-responsive nanoparticles (Arg-CA NPs) in aqueous solution. Following systemic administration, Arg-CA NPs accumulated in inflamed joints of adjuvant-induced arthritis rats. In the acidic synovial microenvironment, the acid-labile imine bonds cleaved, releasing CA. The released CA scavenged intracellular ROS and inhibited NF-κB activation, thereby downregulating pro-inflammatory mediators (e.g., IL-1β, IL-6, TNF-α) and promoting macrophage polarization toward an anti-inflammatory phenotype. This therapeutic process alleviated inflammation and suppressed joint destruction in RA.

## 2. Materials and Methods

### 2.1. Materials

All chemicals were purchased from commercial suppliers and used without further purification. CA (98%) was obtained from J&K Scientific (Beijing, China), and L-Arg was purchased from Sigma–Aldrich (St. Louis, MO, USA). Live/dead staining kits and Cell Counting Kit-8 (CCK-8) were obtained from the Beyotime Institute of Biotechnology(Shanghai, China). Primary antibodies against CD86, CD206, hypoxia-inducible factor-1α (HIF-1α), and cyclooxygenase-2 (COX-2) were purchased from HUABIO (Hangzhou, China), whereas antibodies against p65, phosphorylated p65 (p-p65), phosphorylated IκB-α (p-IκB-α), Arg-1, and iNOS were obtained from ABclonal (Wuhan, China). TNF-α and IL-6 enzyme-linked immunosorbent assay (ELISA) kits were purchased from Invitrogen (Carlsbad, CA, USA).

### 2.2. Synthesis and Characterization of Cinnamaldehyde–Arginine Prodrug (Arg-CA)

Arg (1.74 g, 10 mmol) was dissolved in methanol (100 mL) and stirred at 80 °C for 0.5 h. Under an argon atmosphere, a solution of CA (1.32 g, 10 mmol) in methanol (20 mL) was added dropwise to the Arg solution with continuous stirring. The reaction mixture was then refluxed at 80 °C overnight. After completion, the mixture was filtered to remove unreacted Arg, and the solvent was removed by rotary evaporation. The product was washed three times with cold ethanol (4 °C) to remove unreacted reagents and residual solvent. It was then dried under rotary evaporation overnight to obtain the prodrug (Arg-CA). The reaction yield of Arg-CA was approximately 65–70%. The structure of the product was confirmed by 1H NMR (Bruker BioSpin AG, Fällanden, Switzerland) and ESI-MS (SCIEX, Framingham, MA, USA). The purified Arg-CA was stored at 4 °C and used within the experimental period.

### 2.3. Preparation and Characterization of Arg-CA NPs

Arg-CA powder (50 mg) was dissolved in methanol (1 mL). Subsequently, 200 μL of the Arg-CA methanol solution was slowly added dropwise into 10 mL of deionized water under continuous magnetic stirring. After vigorous stirring for 15 min, the mixture was then transferred into a dialysis bag (MWCO 3500 Da) and dialyzed against deionized water overnight to remove residual methanol and unassembled small molecules. After dialysis, the nanoparticle suspension was collected for further characterization. The concentration of Arg-CA NPs was determined by vacuum freeze-drying a known volume of the nanoparticle suspension and weighing the obtained dry nanoparticles. Based on this method, the concentration of Arg-CA NPs in the suspension was approximately 0.7 mg mL^−1^. The hydrodynamic diameter, polydispersity index (PDI), and zeta potential of Arg-CA NPs were measured at 25 °C using a Zetasizer Nano ZS90 (Malvern Panalytical, Malvern, Worcestershire, UK). The morphology of the nanoparticles was observed by transmission electron microscopy (TEM, Tecnai F20, FEI Company, Hillsboro, OR, USA).

### 2.4. Colloidal Stability

To evaluate colloidal stability, Arg-CA NPs were diluted in H_2_O solution and incubated for 0, 1, 2, and 3 d. At predetermined time points, particle size and PDI were measured using dynamic light scattering (DLS).

### 2.5. In Vitro Drug Release and pH Responsiveness of Arg-CA NPs

The release of Arg from Arg-CA NPs was evaluated using a dialysis method, as commonly reported in nanoparticle drug release studies [23]. Because Arg and CA are linked through an acid-labile Schiff base bond, cleavage of this bond leads to the simultaneous release of both components. Therefore, Arg release was used as an indicator of bond cleavage. Arg-CA NPs (0.2 mL) were placed in a dialysis bag (MWCO = 3.0 kDa) and immersed in phosphate-buffered saline (PBS, pH = 7.4, 6.5, and 5.0, 25 mL) at 37 °C. At predetermined intervals, 100 μL of incubation solution was collected and replaced with an equal volume of fresh PBS. Arg release was measured by ninhydrin reaction as previously reported [24]. Briefly, the extracted buffer reacted with 2% ninhydrin at 80 °C for 15 min. The solution gradually developed a purple color, and absorbance was measured at 570 nm. Concentrations were calculated from a standard curve prepared with free Arg. After mixing Arg-CA NPs with PBS of different pH values for 10 min, take pictures of their visual appearance to evaluate pH responsiveness of Arg-CA NPs.

### 2.6. ABTS+ Assay

Arg-CA NPs (10 and 100 μg mL^−1^) were incubated with ABTS+ solution (7 mM) for 10 min, 30 min, 1 h, 2 h, and 4 h in the dark, and the ABTS radical scavenging activity was evaluated using a previously reported method [25]. Absorbance at 734 nm was measured using a plate reader. The ABTS+ scavenging activity (%) was calculated as follows: ABTS+ scavenging activity (%) = (A0 − Ai)/A0 × 100%, where A0 and Ai represent the absorbance of ABTS+ solution before and after addition of Arg-CA NPs, respectively.

### 2.7. Cell Viability Assay

#### 2.7.1. CCK-8 Assay

Cytotoxicity of Arg-CA NPs was assessed using CCK-8 [26]. RAW264.7 cells were seeded in 96-well plates at 1 × 104 cells/well and incubated at 37 °C with 5% CO_2_ for 24 h. The culture medium was then replaced with DMEM containing graded concentrations of Arg-CA NPs (1.25–80 μg mL^−1^) and incubated at 37 °C with 5% CO_2_ for 24 h. Cell viability was determined according to the manufacturer’s instructions.

#### 2.7.2. Live–Dead Staining

RAW264.7 cells were seeded in 6-well plates at 5 × 105 cells/well and treated with LPS and varying concentrations of Arg-CA NPs (0, 2.5, 5, 10, 20, and 40 μg mL^−1^) for 24 h. After washing with PBS, cells were incubated with Calcein-AM/propidium iodide for 15 min, washed again, and imaged using a fluorescence microscope (DMi8-M, Leica, Germany). Calcein-AM: λex = 495 nm, λem = 515 nm; propidium iodide: λex = 488 nm, λem = 630 nm.

### 2.8. In Vitro Cellular Uptake of Arg-CA NPs

RAW264.7 cells were seeded in laser confocal dishes (NEST, Wuxi, Jiangsu, China). After cell adherence, Coumarin-6-labeled Arg-CA NPs (NPs-C6) or free C6 was added for 1, 4, and 7 h. The cells were fixed with 4% paraformaldehyde for 10 min, stained with DiI (Sigma-Aldrich, St. Louis, MO, USA) for 30 min to label membranes, and counterstained with 4ʹ,6-diamidino-2-phenylindole dihydrochloride (DAPI, Sigma–Aldrich, St. Louis, MO, USA) for 10 min. After washing with PBS, cellular uptake was assessed by flow cytometry (FCM; CytoFLEX LX, Beckman, Brea, CA, USA) and confocal laser scanning microscopy (CLSM, Zeiss, Oberkochen, Germany), as commonly used in nanoparticle uptake studies [27]. NPs-C6: λex = 450 nm, λem = 505 nm.

### 2.9. Mitochondrial Membrane Potential Test

RAW264.7 cells were seeded in 24-well plates and incubated overnight. Cells were left untreated or treated with LPS (100 ng/mL), Arg-CA NPs (10 μg/mL), free CA, or free Arg at equivalent concentrations. Mitochondrial membrane potential (MMP) was assessed using JC-1 dye according to a previously reported method [28]. Cells were incubated with JC-1 dye (20 μM) for 20 min at 37 °C and washed three times with PBS, and fluorescence was observed using a fluorescence microscope.

### 2.10. Intracellular ROS Measurement

Intracellular ROS was detected using the DCFH-DA fluorescent probe according to a previously reported method [29]. RAW264.7 cells were seeded in 12-well plates at 1 × 10^5^ cells/well and incubated overnight. Cells were stimulated with LPS and treated with experimental groups for 24 h. The ROS-sensitive probe DCFH-DA was diluted 1:1000 in serum-free medium to a final concentration of 10 μM and incubated with the cells at 37 °C in the dark for 30 min. Cells were washed three times with PBS to remove excess probe. Intracellular ROS was visualized by CLSM and quantified by FCM. DCFH-DA: λex = 488 nm, λem = 525 nm.

### 2.11. Macrophage Polarization Assay

RAW264.7 cells were seeded in laser confocal dishes at 1 × 104 cells/well and treated with LPS and experimental groups for 24 h. Cells were fixed in 4% paraformaldehyde with 0.1% Triton X-100 (Sigma–Aldrich, St. Louis, MO, USA), blocked with 1% BSA, and incubated with primary antibodies overnight at 4 °C. M1 macrophages were labeled with CD86/iNOS and M2 macrophages with CD206/Arg-1, followed by fluorescent secondary antibodies and DAPI nuclear staining. Images were captured using CLSM.

### 2.12. Establishment of the AIA Model

Healthy female Wistar rats (180–220 g) were obtained from Beijing Vital River Laboratory Animal Technology Co., Ltd (Beijing, China). Animals were housed under SPF conditions and acclimated for 1 week. AIA was induced by subcutaneous injection of 100 μL of complete Freund’s adjuvant containing 10 mg/mL heat-killed mycobacteria (Chondrex, Beijing, China) into the right hind paw [30]. Arthritis progression was monitored daily and was fully established by day 14. All procedures were approved by the Animal Care and Use Committee of Sun Yat-sen University (No. SYSU-IACUC-2024-002629) and conformed to the Laboratory Animal Regulations of Guangdong Province (2010 No. 41).

### 2.13. Biodistribution in AIA Rats

Free DiR and DiR-labeled Arg-CA NPs were intravenously injected into AIA rats to evaluate biodistribution according to previously reported methods [31]. In vivo fluorescence imaging was performed at predetermined time points using an imaging system (IVIS Lumina III, PerkinElmer, Waltham, MA, USA). Fluorescence in regions of interest was analyzed with Living Image Software 4.4 and expressed as average radiant efficiency.

### 2.14. In Vivo Therapeutic Efficacy and Safety Evaluation

Established AIA rats were randomly divided into four groups (*n* = 5). Beginning on day 14 post-immunization, animals received intravenous injections every 3 d for a total of five doses. Groups received saline, free Arg, free CA, or Arg-CA NPs (CA-equivalent dose of 2.5 mg kg^−1^). Free CA and Arg were administered at equimolar doses to those in Arg-CA NPs. The dose of Arg-CA NPs was calculated based on the equivalent CA content (2.5 mg kg^−1^). A group of untreated healthy rats served as normal controls. Arthritis progression was monitored every 3 d by paw swelling and clinical arthritis scores (0–4): 0 = normal; 1 = mild swelling/erythema; 2 = moderate swelling/expansion of erythema; 3 = moderate swelling with extensive erythema; and 4 = severe swelling with pronounced erythema and deformity.

On day 29 after immunization, inflamed joints were harvested for histopathology (H&E, safranin O/fast green, and tartrate-resistant acid phosphatase [TRAP] staining). Paw samples were also analyzed by micro-computed tomography (micro-CT) to assess bone erosion. Micro-CT parameters: voltage 65 kV, current 185 μA, field of view 59.18 × 59.18 mm, and voxel size 50 μm.

Major organs (heart, liver, spleen, lungs, kidneys) were harvested on day 29 for H&E histology. Blood was collected for biochemical analysis of systemic toxicity.

### 2.15. Statistical Analysis

Data are presented as mean ± standard deviation (SD) from three to six independent experiments. Analyses were performed with GraphPad Prism v9. One-way ANOVA and two-way ANOVA with Tukey’s post hoc test were used for group comparisons. Significant differences are defined as * *p* < 0.05, ** *p* < 0.01, *** *p* < 0.001 and **** *p* < 0.0001, and ns means no significance.

## 3. Results

### 3.1. Synthesis and Characterization of Arg-CA NPs

**Figure 1:**
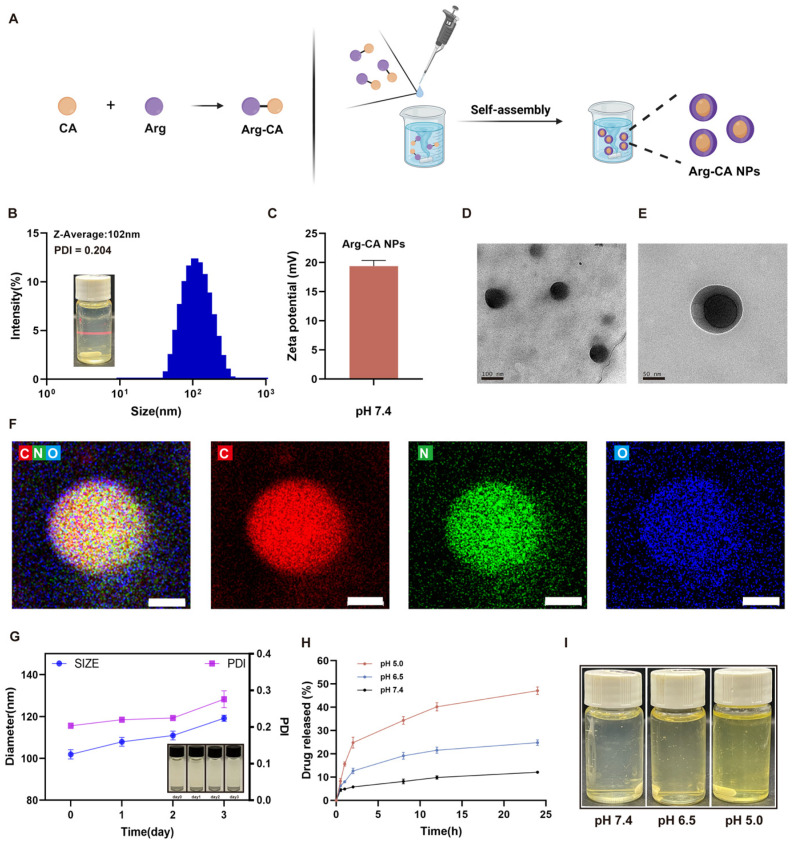
Characterization of Arg-CA nanoparticles (Arg-CA NPs). (**A**) Schematic illustration of Arg-CA synthesis via Schiff base reaction between CA and Arg, followed by self-assembly into pH-responsive nanoparticles. Created in BioRender. Yang, S. (2026). https://BioRender.com/3edbzhj (accessed on 10 march 2026). (**B**) Average hydrodynamic size and PDI of Arg-CA NPs measured by DLS. (**C**) Zeta potential of Arg-CA NPs at pH 7.4. (**D**,**E**) TEM image showing uniform spherical morphology. Scale bar: 100nm and 50 nm. (**F**) Elemental mapping of a single nanoparticle confirming uniform distribution of carbon (C), nitrogen (N), and oxygen (O). Scale bar: 50 nm. (**G**) Particle size and PDI of Arg-CA NPs in aqueous solution over 3 d and photographs of dispersions at different time points. (**H**) Cumulative drug release profile of Arg-CA NPs at different pH values. (**I**) Visual appearance of Arg-CA NPs after incubation under various pH conditions.

The pH-responsive amphiphilic prodrug Arg-CA was synthesized via Schiff base condensation between the aldehyde group of CA and the amino group of Arg. 1H NMR confirmed successful synthesis of Arg-CA, with the characteristic imine bond peaks indicating the presence of the pH-sensitive linkage (Figure S1). ESI-MS analysis further supported the formation of the Arg-CA conjugate. The spectrum showed a characteristic peak at *m*/*z* ≈ 289 corresponding to the protonated molecular ion [M+H]^+^, which is consistent with the expected molecular weight of Arg-CA (Figure S2). The self-assembly behavior of Arg-CA was verified by nanoprecipitation, yielding well-dispersed nanoparticles (Figure 1A).

DLS showed that Arg-CA NPs had an average hydrodynamic diameter of ~102 nm with a narrow distribution (PDI = 0.204) (Figure 1B). The zeta potential was +19 mV, favoring interaction with negatively charged inflamed cell membranes (Figure 1C). TEM confirmed a uniform spherical morphology of ~100 nm (Figure 1D), consistent with the DLS results. Previous studies have suggested that spherical morphology and nanoscale dimensions facilitate cellular internalization [32]. Elemental mapping further demonstrated the uniform distribution of C, N, and O within the nanoparticles (Figure 1E). The colloidal stability of Arg-CA NPs in aqueous solution was evaluated by monitoring particle size and PDI over 3 d. No significant changes were observed (Figure 1F), indicating excellent colloidal stability.

Because RA joints exhibit an acidic microenvironment, pH-responsive carriers are considered highly promising for RA therapy [33,34]. Unlike conventional nanocarriers that often require complex chemical modification to achieve acid sensitivity, Arg-CA NPs self-assemble through Schiff base formation, providing a straightforward preparation route and controllable structure. Under acidic conditions mimicking RA joints, Arg-CA NPs underwent acid-triggered disassembly and drug release (Figure 1G). However, under physiological pH (7.4), only minimal release was observed, indicating that the Schiff base linkage remains relatively stable under near-neutral conditions.

Collectively, these results demonstrate that Arg-CA NPs possess well-defined physicochemical properties, excellent stability, and pH-responsive release behavior, making them well suited for systemic delivery and targeted drug release in RA joints.

### 3.2. Cellular Internalization and Cytocompatibility of Arg-CA NPs

**Figure 2:**
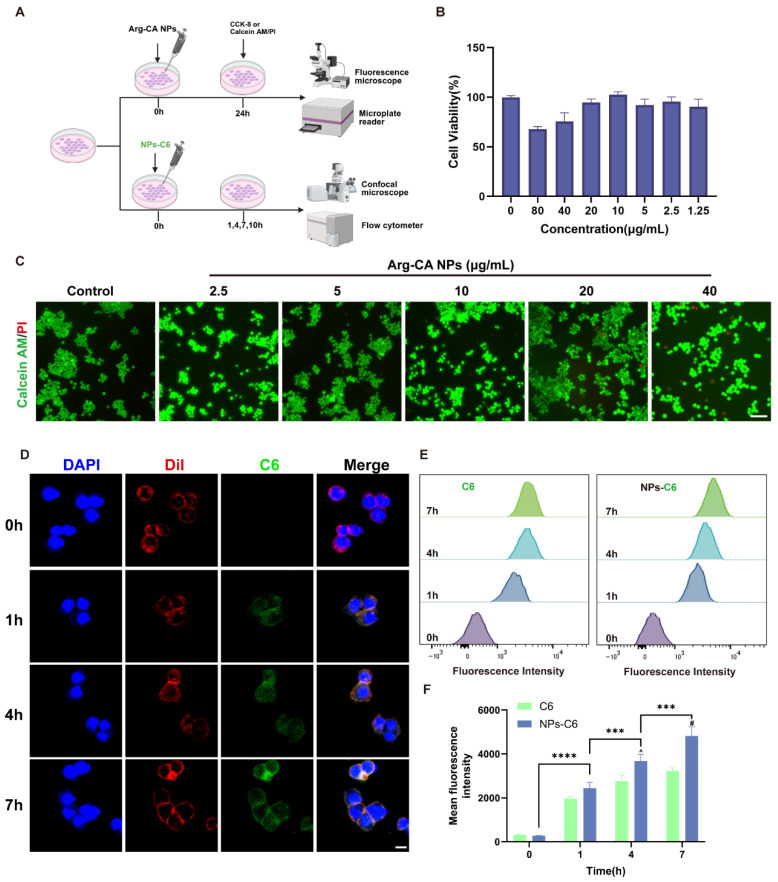
Cell viability and cellular uptake of Arg-CA NPs in vitro. (**A**) Schematic illustration of assays for cytocompatibility and cellular uptake. Created in BioRender. Yang, S. (2026). https://BioRender.com/6nxygjd (accessed on 10 march 2026). (**B**) CCK-8 assay of RAW264.7 cells treated with different concentrations of Arg-CA NPs for 24 h. (**C**) Live/dead staining of RAW264.7 cells after 24 h treatment with Arg-CA NPs at different concentrations. (**D**) CLSM images of RAW264.7 cells incubated with NPs-C6 at 1, 4, and 7 h. Green: NPs-C6; red: cell membrane (DiI); blue: nuclei (DAPI). Scale bar: 10 μm. (**E**) Flow cytometry (FCM) analysis of C6 fluorescence intensity in RAW264.7 cells treated with free C6 or C6-labeled Arg-CA NPs at 0, 1, 4, and 7 h. (**F**) Quantitative analysis of mean fluorescence intensity by FCM. Data are presented as mean ± SD (*n* = 3). Statistical significance between every 2 groups was calculated via a one-way ANOVA test and two-way ANOVA test: *** *p* < 0.001, **** *p* < 0.0001, ^^^ *p* < 0.01 vs. 4 h C6 group; ^#^ *p* < 0.0001 vs. 7 h C6 group.

CLSM and complementary assays were employed to assess the biosafety and cellular uptake of Arg-CA NPs in vitro (Figure 2A). The CCK-8 assay showed negligible cytotoxicity at concentrations ≤10 μg mL^−1^ (Figure 2B). Live/dead staining further confirmed biocompatibility, with nearly all cells exhibiting green fluorescence (viable cells) after 24 h incubation across different concentrations (Figure 2C and Figure S3). Red fluorescence (dead cells) appeared only at concentrations ≥20 μg mL^−1^. In addition, Arg-CA NPs exhibited a hemolysis rate below 1% at all tested concentrations (Figures S4 and S5). Based on these results, 10 μg mL^−1^ was selected for subsequent experiments.

Cellular internalization is critical for nanoparticle-based therapies, as endocytosis enables delivery of therapeutic payloads to intracellular targets [35,36]. To evaluate uptake, NPs-C6 were incubated with RAW264.7 cells for different durations. CLSM imaging revealed progressive cytoplasmic green fluorescence over time, confirming time-dependent internalization of Arg-CA NPs (Figure 2D). FCM analysis corroborated this trend, showing steadily increasing fluorescence intensity (Figure 2E,F). To compare uptake efficiency with free molecules, RAW264.7 cells were treated with free C6 under identical conditions. NPs-C6 displayed markedly higher cellular uptake than free C6, indicating enhanced transport efficiency of the nanoparticle formulation. These findings demonstrated that Arg-CA NPs combine favorable cytocompatibility with efficient macrophage internalization, providing a strong foundation for their therapeutic potential in RA.

### 3.3. Arg-CA NPs Alleviate Oxidative Stress in Macrophages

**Figure 3:**
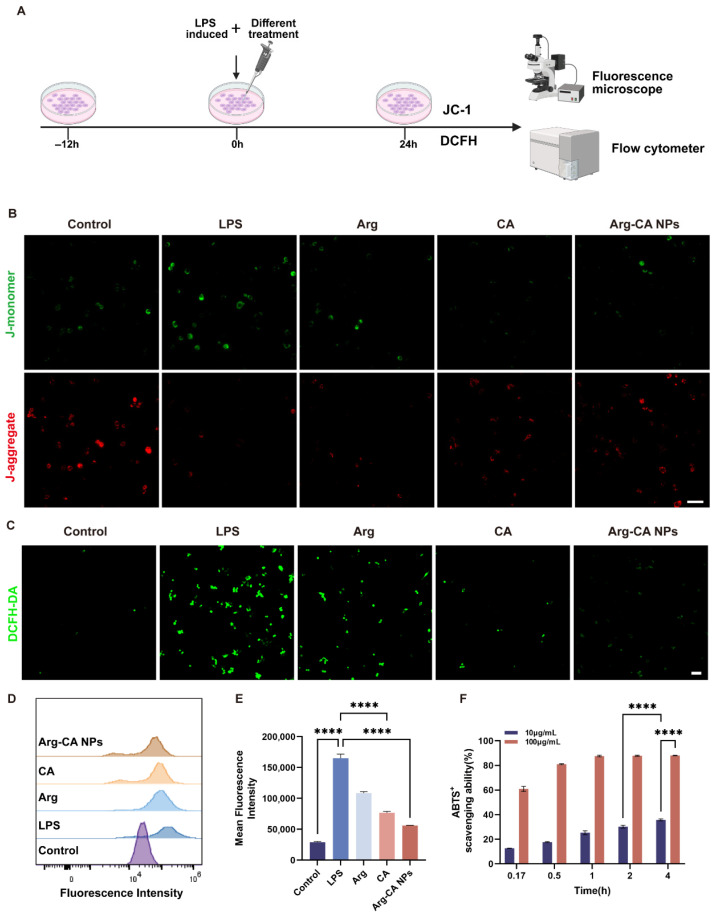
Antioxidant activity of Arg-CA NPs in vitro. (**A**) Schematic illustration of assays evaluating the antioxidant capacity of Arg-CA NPs. Created in BioRender. Yang, S. (2026). https://BioRender.com/xuzntqu (accessed on 10 march 2026). (**B**) JC-1 staining of RAW264.7 cells after different treatments, showing mitochondrial membrane potential. Red: J-aggregates; green: J-monomers. Scale bar: 50 μm. (**C**) Fluorescence images of intracellular ROS in RAW264.7 cells using DCFH-DA (green). Scale bar: 50 μm. (**D**) FCM analysis of intracellular ROS levels. (**E**) Quantification of mean fluorescence intensity from (D). (**F**) ABTS+ radical scavenging activity of Arg-CA NPs at different concentrations. Data are presented as mean ± SD (*n* = 3). Statistical significance between every 2 groups was calculated via a one-way ANOVA test: **** *p* < 0.0001.

To assess the antioxidant capacity of Arg-CA NPs, RAW264.7 cells were treated under different conditions for 24 h (Figure 3A). MMP is a key indicator of mitochondrial function and cellular energy metabolism. In RA, excessive oxidative stress contributes to synovial inflammation and joint destruction, with mitochondria serving as primary targets of ROS. Sustained oxidative stress disrupts mitochondrial integrity, leading to decreased MMP, which is recognized as an early hallmark of mitochondrial dysfunction and apoptosis [37]. MMP was evaluated using the JC-1 probe. In untreated cells, high membrane potential promoted JC-1 aggregation in the mitochondrial matrix, generating strong red fluorescence (Figure 3B). Upon LPS stimulation, J-aggregates were converted to monomers, producing strong green fluorescence that indicated MMP loss. In contrast, treatment with Arg-CA NPs largely preserved red fluorescence, demonstrating protection of mitochondrial integrity and prevention of MMP decline.

ROS scavenging was further assessed using the probe DCFH-DA. Fluorescence microscopy showed that LPS stimulation induced strong green fluorescence, reflecting elevated ROS levels. Treatment with Arg-CA NPs markedly reduced fluorescence intensity, suggesting superior ROS elimination compared with other groups (Figure 3C). FCM confirmed this effect, with a significant reduction in mean fluorescence intensity relative to the LPS group (Figure 3D,E).

To validate intrinsic antioxidant potential, the ABTS+ radical scavenging assay was performed. Arg-CA NPs exhibited concentration- and time-dependent scavenging activity, achieving substantial radical clearance even at low concentrations (Figure 3F). Prior studies indicate that the nitrogen atom in the –C=N– imine group acts as an electron donor, promoting dissociation of nearby labile hydrogens and facilitating hydrogen transfer, thereby enhancing free radical scavenging [38]. The observed antioxidant activity of Arg-CA NPs is therefore likely attributable to electron transfer within the imine bond.

Together, these findings demonstrate that Arg-CA NPs suppress intracellular ROS accumulation and preserve mitochondrial integrity under inflammatory stress, highlighting their potent antioxidative function in macrophages.

### 3.4. Regulation of Macrophage Polarization by Arg-CA NPs

**Figure 4:**
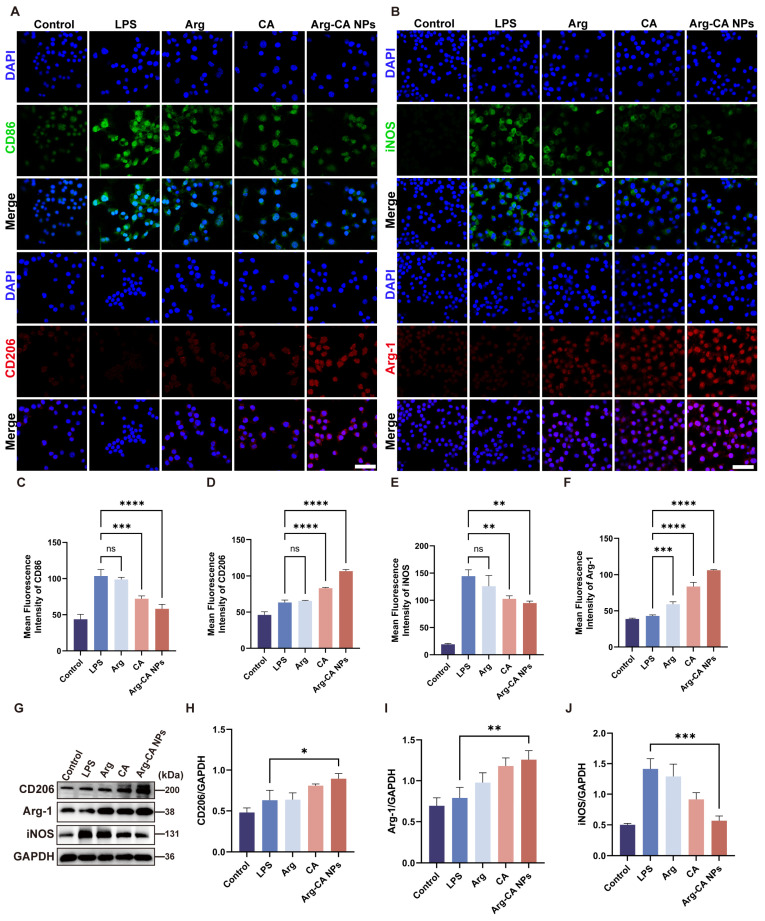
Arg-CA NPs regulate macrophage polarization in vitro. (**A**) CLSM images of RAW264.7 macrophages stained with CD86 (green, M1 marker) and CD206 (red, M2 marker). Scale bar: 50 μm. (**B**) CLSM images of cells stained with iNOS (green, M1 marker) and Arg-1 (red, M2 marker). Scale bar: 50 μm. (**C**–**F**) Semi-quantitative fluorescence intensity of CD86, CD206, iNOS, and Arg-1. (**G**) Western blot analysis of M1/M2 markers in RAW264.7 cells after different treatments. (**H**–**J**) Quantitative protein expression of CD206, Arg-1, and iNOS normalized to GAPDH. Data are presented as mean ± SD (*n* = 3). Statistical significance between every 2 groups was calculated via a one-way ANOVA test: ns means no significance, * *p* < 0.05, ** *p* < 0.01, *** *p* < 0.001, **** *p* < 0.0001.

Macrophages play a central role in RA pathogenesis [39]. Pro-inflammatory M1 macrophages exacerbate tissue injury, whereas anti-inflammatory M2 macrophages secrete cytokines such as IL-10 and TGF-β that promote inflammation resolution and tissue repair [40]. To assess the effects of Arg-CA NPs on macrophage polarization, CD86 and CD206 were employed as immunofluorescence markers for M1 and M2 phenotypes, respectively. As shown in Figure 4A, LPS-stimulated RAW264.7 cells exhibited elevated CD86 fluorescence compared with controls, indicating M1 polarization. Treatment with Arg-CA NPs markedly reduced CD86 fluorescence and enhanced CD206 fluorescence. Additional markers, iNOS (M1) and Arg-1 (M2), were examined by immunofluorescence. Arg-CA NPs suppressed LPS-induced iNOS expression while upregulating Arg-1 expression (Figure 4B). Semi-quantitative fluorescence analysis further confirmed that Arg-CA NPs decreased CD86 and iNOS while increasing CD206 and Arg-1 relative to the LPS group (Figure 4C–F).

Western blotting further assessed M1/M2 polarization markers. LPS stimulation significantly increased iNOS protein levels (Figure 4G). Arg-CA NPs downregulated iNOS while upregulating Arg-1 and CD206, consistent with immunofluorescence findings. Densitometric quantification corroborated these results (Figure 4H–J). Together, these data indicate that Arg-CA NPs effectively inhibit M1 polarization while promoting M2 polarization, thereby contributing to resolution of inflammation.

### 3.5. Anti-Inflammatory Mechanisms of Arg-CA NPs

**Figure 5:**
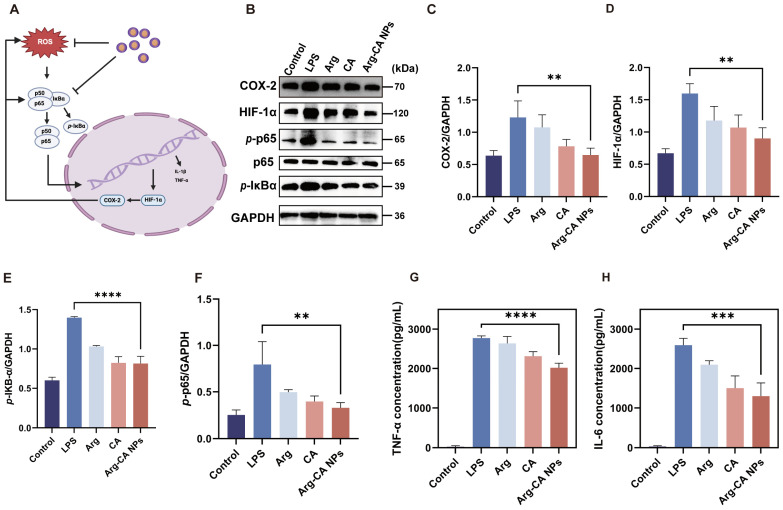
Arg-CA NPs suppress inflammatory responses in vitro. (**A**) Mechanism of Arg-CA NPs in regulating inflammatory signaling pathways in rheumatoid arthritis. Created in BioRender. Yang, S. (2026). https://BioRender.com/m5j27cy (accessed on 10 march 2026). (**B**) Western blot analysis of COX-2, HIF-1α, and NF-κB pathway proteins in RAW264.7 cells after different treatments. (**C**–**F**) Quantification of protein expression normalized to GAPDH. (**G**,**H**) ELISA quantification of TNF-α and IL-6 secretion in RAW264.7 cells treated with different groups. Data are presented as mean ± SD (*n* = 3). Statistical significance between every 2 groups was calculated via a one-way ANOVA test: ** *p* < 0.01, *** *p* < 0.001, **** *p* < 0.0001.

To further elucidate the anti-inflammatory mechanisms of Arg-CA NPs, Western blotting was performed to evaluate the expression of key inflammatory regulators. During inflammation, cellular stimulation induces upregulation of cyclooxygenase-2 (COX-2) by mediators such as interleukin-1 (IL-1) and TNF-α [41]. Excessive expression of HIF-1α further promotes angiogenesis, immune cell activation, and secretion of pro-inflammatory mediators [9]. Accordingly, COX-2 and HIF-1α expression was examined as an indicator of inflammatory activity. LPS stimulation markedly increased both proteins (Figure 5A), whereas Arg-CA NPs significantly downregulated their expression. Proteins associated with the NF-κB signaling pathway, including p65, p-p65, and p-IκB-α, were also assessed. LPS stimulation enhanced phosphorylation of p65 and IκB-α, indicating NF-κB pathway activation. In contrast, Arg-CA NPs reduced phosphorylation levels, suggesting inhibition of NF-κB signaling. Quantitative immunoblotting analysis corroborated these findings (Figure 5B–E).

As downstream mediators, TNF-α and IL-6 play critical roles in sustaining chronic inflammation and driving joint destruction [42]. Their secretion was quantified by ELISA. Consistent with the immunoblotting results, Arg-CA NPs markedly decreased TNF-α and IL-6 levels in activated macrophages (Figure 5F,G), demonstrating their ability to suppress pro-inflammatory cytokine release. Overall, these results indicate that Arg-CA NPs modulate macrophage polarization and attenuate inflammatory responses by downregulating COX-2, HIF-1α, and NF-κB signaling while reducing the secretion of TNF-α and IL-6.

### 3.6. In Vivo Biodistribution and Biocompatibility of Arg-CA NPs

**Figure 6:**
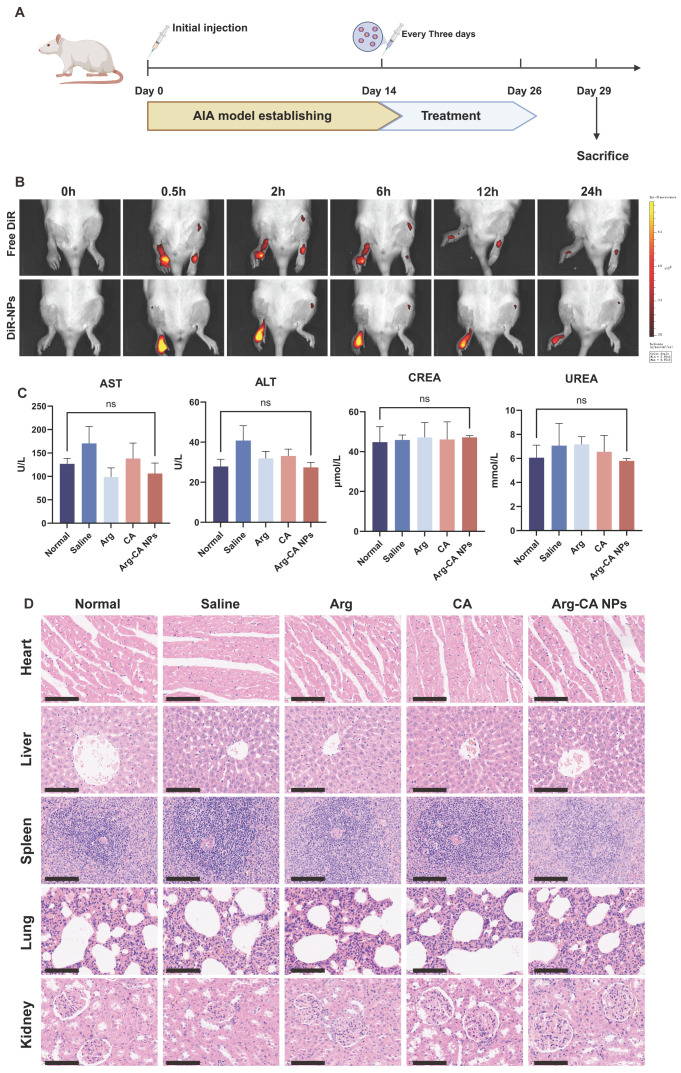
In vivo biodistribution and biocompatibility of Arg-CA NPs. (**A**) Experimental scheme for in vivo evaluation. Created in BioRender. Yang, S. (2026). https://BioRender.com/v5nttbj (accessed on 10 march 2026). (**B**) Time-course fluorescence imaging of AIA rats after intravenous injection of free DiR or DiR-labeled Arg-CA NPs. (**C**) Serum biochemical parameters of AIA rats on day 29 after treatment with Arg-CA NPs. (**D**) Representative H&E-stained sections of major organs (heart, liver, spleen, lung, kidney) from AIA rats on day 29. Scale bar: 50 μm. ALT, alanine aminotransferase; AST, aspartate aminotransferase; CREA, creatinine. Data are presented as mean ± SD (*n* = 3). Statistical analysis was performed using one-way ANOVA; ns indicates no significant difference.

Given the strong anti-inflammatory effects of Arg-CA NPs observed in vitro, their in vivo performance was evaluated using AIA rat models (Figure 6A). In RA, the inflammatory microenvironment is characterized by vascular proliferation and dilation around affected joints. Nanoparticles can traverse the enlarged vascular gaps and accumulate at inflamed sites via inflammatory cell mediation, a process known as the ELVIS effect [43,44]. To investigate whether Arg-CA NPs could exploit this effect for targeted accumulation, biodistribution was monitored by near-infrared fluorescence imaging. DiR-labeled Arg-CA NPs (DiR-NPs) or free DiR was intravenously administered to AIA rats, and whole-body fluorescence imaging was used to track distribution. In the free DiR group, fluorescence appeared in the inflamed right paw within 0.5 h but rapidly declined after 6 h, reflecting fast systemic clearance. In contrast, the DiR-NP group displayed strong fluorescence in the inflamed paw that persisted for 12 h and remained detectable at 24 h (Figure 6B). These results demonstrate that Arg-CA NPs effectively exploit the ELVIS effect for preferential accumulation and prolonged retention in arthritic joints.

Systemic toxicity was evaluated by serum biochemistry. Compared with AIA controls, rats treated with Arg-CA NPs showed no significant changes in liver function markers (ALT, AST) or kidney function markers (UREA, BUN) (Figure 6C), indicating minimal hepatic and renal burden. Histological analysis by H&E staining further confirmed the absence of pathological alterations in major organs. The heart displayed intact architecture without inflammatory infiltration; the liver retained normal lobular morphology with preserved hepatocyte cords; the spleen exhibited intact architecture with clearly demarcated white and red pulp; the lung showed normal alveolar structure without edema or inflammation; and the kidney revealed intact glomeruli without evidence of congestion or edema (Figure 6D). Collectively, these findings indicate that Arg-CA NPs possess excellent biocompatibility and do not induce detectable systemic toxicity, supporting their potential for safe therapeutic application in RA.

### 3.7. Therapeutic Efficacy of Arg-CA NPs in AIA Rats

**Figure 7:**
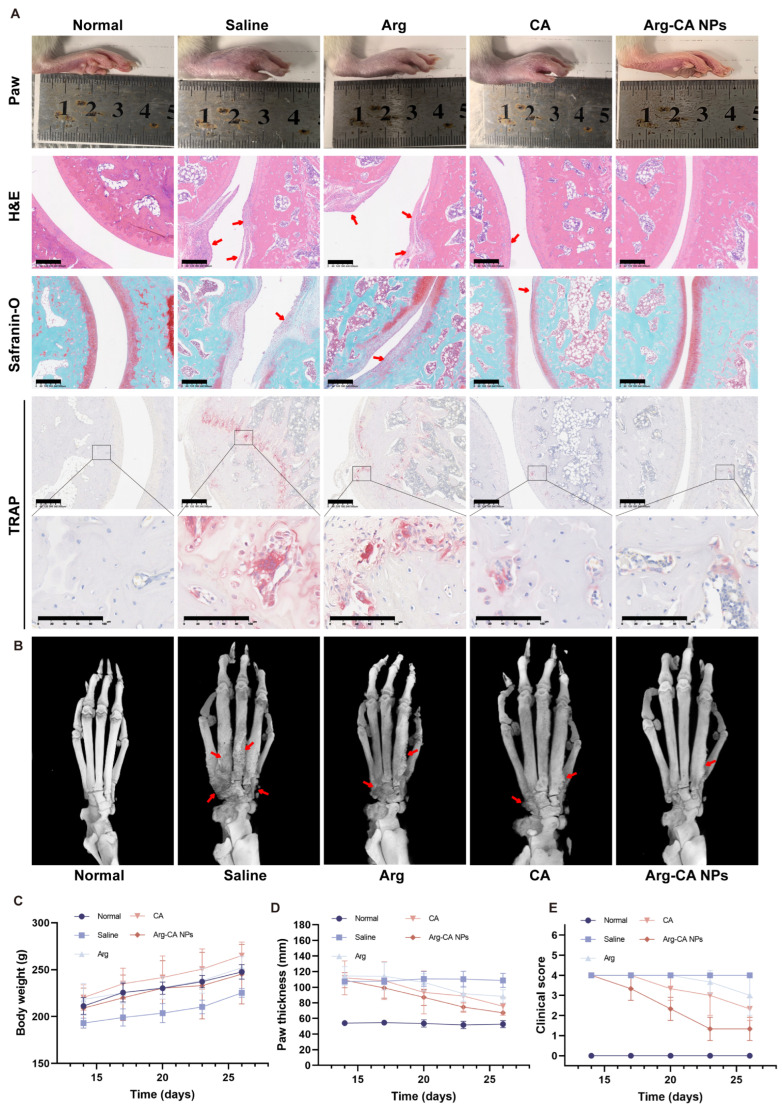
Therapeutic efficacy of Arg-CA NPs in AIA rats. (**A**) Representative images of hind paw morphology and histological sections of ankle joints after different treatments (H&E staining, safranin O/fast green staining, TRAP staining). Scale bar: 300 μm and 100 μm. The red arrow represents the lesion site. The square indicates the selected region of interest (ROI), which is shown at higher magnification below. (**B**) Representative micro-CT images of ankle joints. (**C**–**E**) Clinical assessment of arthritis progression in AIA rats after different treatments: (**C**) body weight, (**D**) hind paw thickness, and (**E**) clinical scores.

The AIA rat model was employed to evaluate the in vivo therapeutic efficacy of Arg-CA NPs. Animals were randomly assigned to different experimental groups. Beginning on day 14 after immunization, rats received intravenous injections of saline, free CA, free Arg, or Arg-CA NPs (CA-equivalent dose of 2.5 mg kg^−1^) every 3 d for a total of five administrations; untreated healthy rats served as normal controls (Figure 6A). Representative images of hind paws and histological sections from all groups are shown in Figure 7A. Macroscopic examination revealed severe swelling in the hind paws of saline-treated AIA rats. Treatment with free Arg or CA led to partial reduction in swelling, whereas Arg-CA NPs produced a more pronounced improvement.

RA-induced inflammation disrupts cartilage via excessive pro-inflammatory cytokines and matrix metalloproteinases. Overactive osteoclasts further resorb subchondral bone, exacerbating cartilage erosion and accelerating joint destruction [45,46]. Histological analysis confirmed these pathological features. H&E staining revealed severe synovial hyperplasia and inflammatory infiltration in saline-, CA-, and Arg-treated groups, whereas Arg-CA NPs significantly alleviated these changes. Safranin O–fast green staining showed that the cartilage layer was largely preserved in the Arg-CA NP group, in contrast to obvious erosion in other groups. TRAP staining further demonstrated reduced osteoclast number and activity in the Arg-CA NP group, indicating effective suppression of bone resorption.

Bone erosion was further evaluated using micro-CT (Figure 7B). Saline-treated rats exhibited marked ankle bone loss and rough phalangeal surfaces. Although Arg-CA NP-treated joints displayed some surface irregularities, bone architecture and joint morphology were largely preserved compared with other groups.

Clinical indicators, including body weight, paw thickness, and arthritis scores, were monitored. Saline-treated rats exhibited slower body weight gain relative to the normal group. Rats treated with free CA, free Arg, or Arg-CA NPs displayed body weight trends similar to normal controls. Regarding arthritis progression, saline-treated rats showed severe paw swelling and high clinical scores, whereas free CA and Arg provided only modest improvements. In contrast, Arg-CA NPs significantly reduced paw thickness and arthritis scores, confirming superior therapeutic efficacy (Figure 7C–E). Collectively, these findings demonstrate that Arg-CA NPs effectively attenuate joint inflammation, protect cartilage, suppress osteoclast activity, and preserve bone structure in AIA rats, thereby alleviating both inflammatory and structural hallmarks of RA.

## 4. Conclusions

In this study, Arg-CA NPs with excellent aqueous dispersibility, physiological stability, and biocompatibility were developed through a Schiff base reaction and self-assembly of CA and Arg. The imine linkage conferred acid-triggered degradation, enabling selective release of CA and Arg in the mildly acidic inflammatory microenvironment of RA. In vitro, Arg-CA NPs effectively scavenged intracellular ROS, preserved mitochondrial integrity, suppressed NF-κB activation, and reprogrammed macrophages toward an anti-inflammatory phenotype, thereby ameliorating the inflammatory microenvironment. In vivo, Arg-CA NPs accumulated in inflamed joints via the ELVIS effect and significantly reduced joint swelling, synovial inflammation, cartilage erosion, and bone destruction, while exhibiting minimal systemic toxicity.

However, the present work primarily focused on evaluating the antioxidant and anti-inflammatory effects of the nanoformulation; the detailed intracellular metabolic processes and long-term pharmacokinetic behavior of Arg-CA NPs have not yet been systematically investigated. In addition, the biological effects of CA and Arg were evaluated individually to compare their activity with that of Arg-CA NPs. A combined treatment of free CA and free Arg was not included, which may provide further insight into their potential synergistic effects. Further investigations addressing the above limitations may support the development of Arg-CA NPs as a promising and safe therapeutic strategy for RA and provide new insights into nanomedicine-based interventions for inflammation-driven diseases.

## Data Availability

The original contributions presented in this study are included in the article/Supplementary Materials. Further inquiries can be directed to the corresponding authors.

## Supplementary Materials

The following supporting information can be downloaded at https://www.mdpi.com/article/10.3390/antiox15040469/s1. Figure S1: ^1^H NMR spectrum of Arg-CA confirming successful conjugation between CA and Arg; Figure S2: ESI-MS spectrum of the Arg-CA conjugate recorded in positive ion mode; Figure S3: Live/dead staining of RAW264.7 cells treated with various concentrations of Arg-CA NPs for 24 h; Figure S4: Representative image of hemolysis assay; Figure S5: Hemolysis ratio at different concentrations. Results were expressed as mean ± SD. (ns represented not significant, *n* = 3).
