# Supplementary material for: pH-Responsive Cinnamaldehyde–Arginine Nanoprodrug for Targeted Rheumatoid Arthritis Therapy via Antioxidant Activity and Macrophage Reprogramming
Source: Antioxidants (Basel). 2026 Apr 10;15(4):469. doi: 10.3390/antiox15040469 (PMC13114153; doi:10.3390/antiox15040469)
Supplement: Supplementary file 1 [file antioxidants-15-00469-s001.zip › antioxidants-4168457-supplementary.pdf]

Article

pH-Responsive Cinnamaldehyde–Arginine Nanoprodrug for Targeted Rheumatoid Arthritis Therapy via Antioxidant Activity and Macrophage Reprogramming

Lihong Huang [1,2,†], Wenlong Zhang [1,†], Shuai Qiu [1], Dazhi Yang [3], Qingyun Tang [1], Jiajun Huang [1], Lei Liu [1], Yang Kang [2,*] and Shuo Tang [1,*]

1 Department of Orthopaedics, The Eighth Affiliated Hospital, Sun Yat-Sen University, Shenzhen 518033, China; huanglh65@mail2.sysu.edu.cn (L.H.); chmlung@mail2.sysu.edu.cn (W.Z.); qiush23@mail.sysu.edu.cn (S.Q.); tangqy6@mail2.sysu.edu.cn (Q.T.); huangjj266@mail.sysu.edu.cn (J.H.); liulei56@mail.sysu.edu.cn (L.L.)
2 Scientific Research Center, The Seventh Affiliated Hospital, Sun Yat-Sen University, Shenzhen 518107, China
3 Department of Spine Surgery, Shenzhen Nanshan Hospital, Shenzhen 518052, China; dazhiyang@email.szu.edu.cn
* Correspondence: kangy26@mail.sysu.edu.cn (Y.K.); tangsh35@mail.sysu.edu.cn (S.T.)
† These authors contributed equally to this work.

Experimental Section

Cell culturing and treatment

RAW264.7 cells were cultured in Dulbecco's Modified Eagle's Medium (DMEM) supplemented with 10% fetal bovine serum and 1% penicillin–streptomycin at 37 °C in a humidified atmosphere containing 5% CO_2. The medium was replaced every 2–3 days to maintain optimal cell growth. Inflammatory polarization was induced by stimulation with lipopolysaccharide (LPS, 100 ng/mL).

Hemolysis Assay

Whole blood was collected from healthy Wistar rats via venipuncture into anticoagulant tubes containing either EDTA or sodium citrate. The samples were gently inverted to mix and centrifuged at 3000 rpm for 15 min to obtain red blood cells (RBCs). Various concentrations of Arg-CA NPs (80, 40, 20, 10, 5, 2.5 and 1.25 μg/mL) were prepared in PBS (pH 7.4). For each group, 1 mL of the nanoparticle solution, ultrapure water (positive control), or PBS (negative control) was mixed with 20 μL of RBC suspension. The mixtures were incubated at 37 °C for 4 h, followed by centrifugation at 3000 rpm for 15 min. The supernatants were visually assessed for hemolysis, and photographs were taken under identical lighting conditions using a smartphone to document color differences. Subsequently, the supernatants were transferred to a 96-well plate, and the absorbance at 540 nm was measured using a microplate reader (BioTek, USA). The hemolysis percentage was calculated using the following formula:

$$\boldsymbol{Hemolysis}(\%) = \frac{\boldsymbol{A_{sample}} - \boldsymbol{A_{PBS}}}{\boldsymbol{A_{H_2O}} - \boldsymbol{A_{PBS}}} \times \mathbf{100\%}$$

where A_{sample}, A_{PBS} and A_{H2O} correspond to the absorbance values of the test sample, PBS, and ultrapure water groups, respectively.

Western blotting analysis

The RAW 264.7 cells were seeded in 6-well plates at a density of 3×10^5 cells per well and incubated overnight. The cells were then stimulated with LPS and treated with various experimental groups for 24 h. Total cellular proteins were extracted on ice using a radioimmunoprecipitation assay lysis buffer supplemented with loading buffer and a phosphatase inhibitor cocktail. Protein samples were separated using SDS-PAGE and then transferred onto polyvinylidene difluoride (PVDF) membranes. The PVDF membranes were blocked with 5% skim milk for 1 h and subsequently incubated with specific primary antibodies overnight at 4°C. Upon washing with PBST, the membranes were incubated with appropriate secondary antibodies at room temperature for 1 h. Finally, the protein bands were visualised using an Omni-ECL™ Femto Light Chemiluminescence Kit (Vazyme, China) and imaged using a ChemiDoc imaging system (Bio-Rad).

ELISA assay

RAW264.7 cells were seeded at a density of 3×10^5 cells/well in 12-well plates and incubated overnight. The cells were then treated with LPS in combination with various formulations for 24 h. Following incubation, the culture supernatants were collected via centrifugation at 12,000 rpm for 5 min. The levels of TNF-α and IL-6 in the supernatants were quantified using a mouse TNF-α High Sensitivity ELISA Kit (EK201BHSS, Multi Sciences) and a mouse IL-6 ELISA kit (EK2236-01, Multi Sciences) by following manufacturer's instructions.

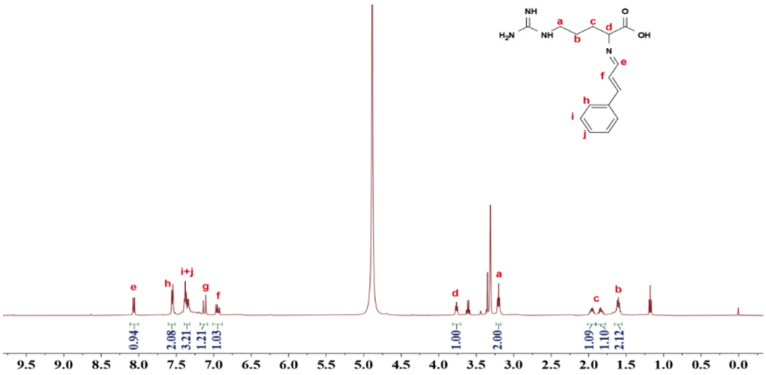

Figure S1. ^{1}H NMR spectrum of Arg-CA confirming successful conjugation between CA and Arg.

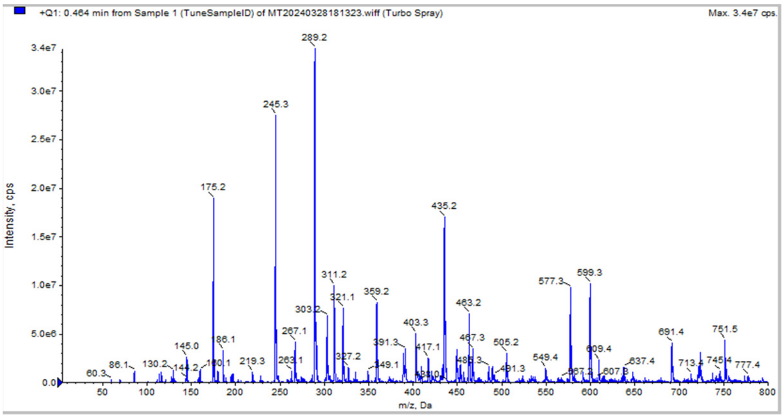

Figure S2.ESI-MS spectrum of the Arg-CA conjugate recorded in positive ion mode.

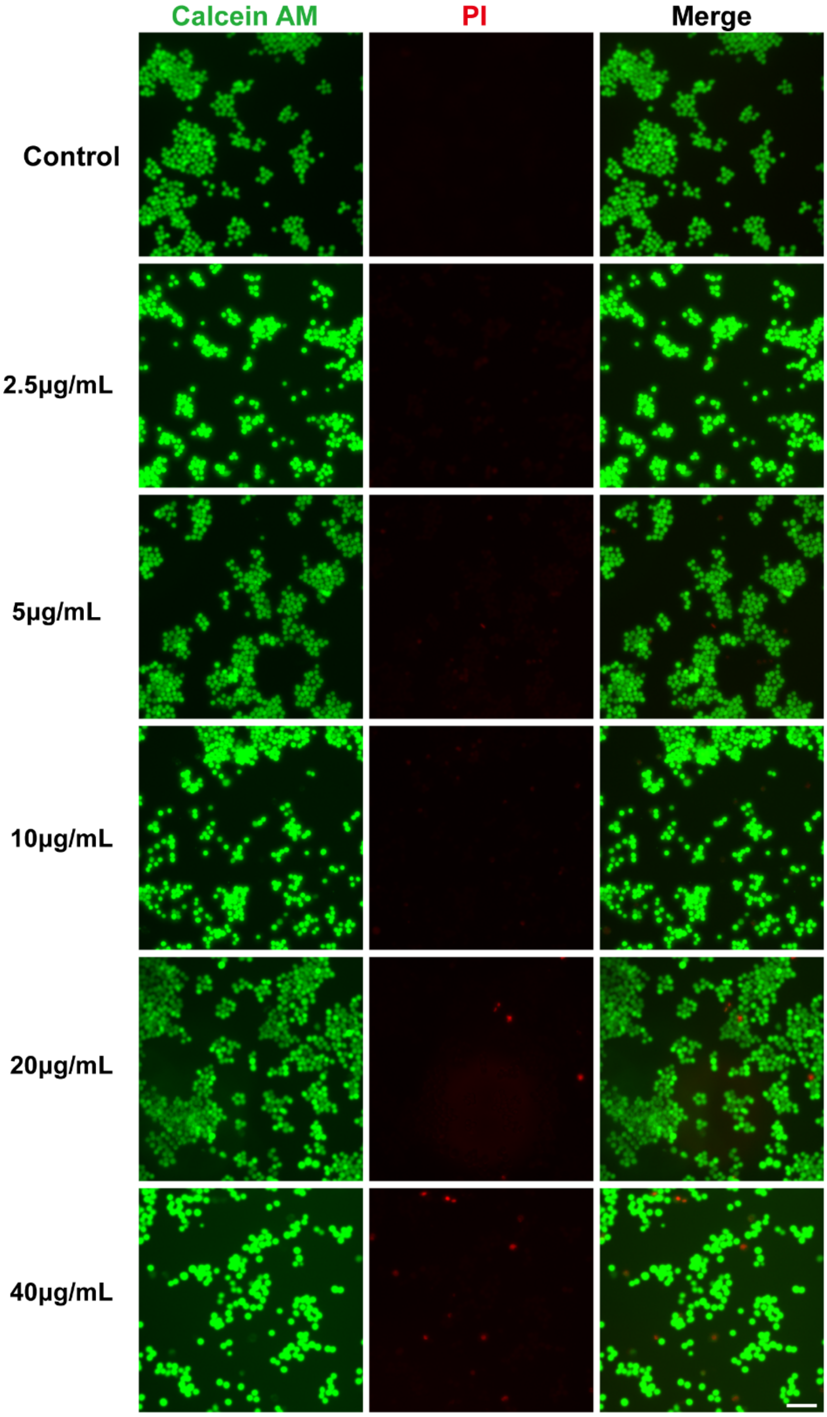

Figure S3. Live/dead staining of RAW264.7 cells treated with various concentrations of Arg-CA NPs for 24 h.

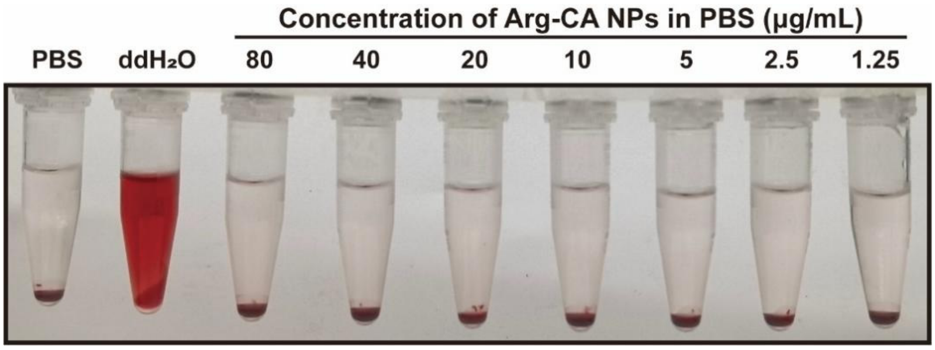

Figure S4. Representative image of hemolysis assay.

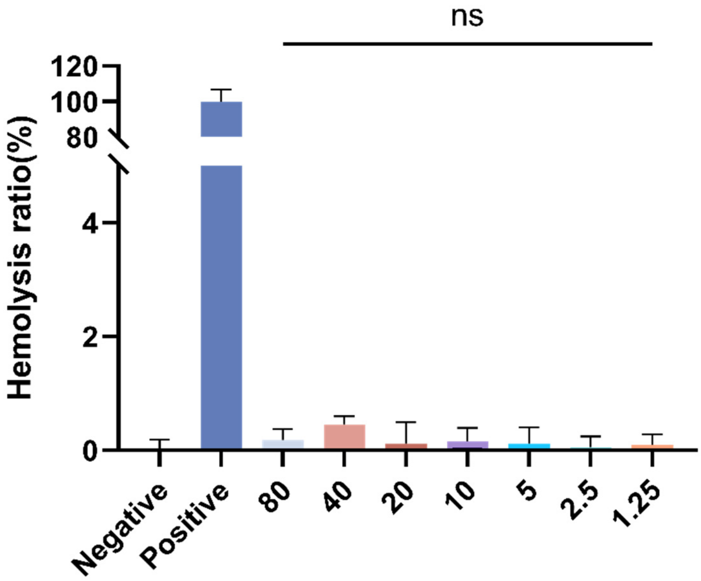

Figure S5. Hemolysis ratio at different concentrations. Results were expressed as mean ± SD. (ns represented not significant, $n = 3$).
